# Stimulation of Treg Cells to Inhibit Osteoclastogenesis in Gorham-Stout Disease

**DOI:** 10.3389/fcell.2021.706596

**Published:** 2021-08-27

**Authors:** Michela Rossi, Ippolita Rana, Paola Sabrina Buonuomo, Giulia Battafarano, Viviana De Martino, Matteo D’Agostini, Ottavia Porzio, Cristiana Cipriani, Salvatore Minisola, Rita De Vito, Davide Vecchio, Michaela Veronika Gonfiantini, Alessandro Jenkner, Andrea Bartuli, Andrea Del Fattore

**Affiliations:** ^1^Bone Physiopathology Research Unit, Genetics and Rare Diseases Research Division, Bambino Gesù Children’s Hospital, IRCCS, Rome, Italy; ^2^Rare Diseases and Medical Genetic Unit, Bambino Gesù Children’s Hospital, IRCCS, Rome, Italy; ^3^Department of Clinical, Internal, Anaesthesiological and Cardiovascular Sciences, Sapienza University, Rome, Italy; ^4^Clinical Laboratory, Bambino Gesù Children’s Hospital, IRCCS, Rome, Italy; ^5^Department of Experimental Medicine, University of Rome Tor Vergata, Rome, Italy; ^6^Department of Histopathology, Bambino Gesù Children’s Hospital, IRCCS, Rome, Italy; ^7^Division of Immunology and Infectious Diseases, Department of Pediatrics, Bambino Gesù Children’s Hospital, IRCCS, Rome, Italy

**Keywords:** diseases and disorders of/related to bone, osteoclasts, osteoimmunology, regulatory T cells, Gorham-Stout disease

## Abstract

Gorham-Stout disease (GSD) is a very rare syndrome displaying excessive bone erosion and vascular lesion. Due to the rarity of the disease and to the limited studies, its etiopathogenesis is not entirely known. The involvement of immune system in the progressive osteolysis was recently suggested. Indeed, extensive reciprocal interactions between the immune and skeletal systems have been demonstrated. This study aimed to evaluate alterations of immune cells in GSD. An increase of CD8+ cells and reduction of CD4+ and CD4+CD25+CD127^low^ cells was revealed in patients. Interestingly, patients’ regulatory T cells maintain the ability to respond to extracellular stimuli and to regulate osteoclastogenesis; GSD cells proliferate under aCD3/CD28 signal reaching similar levels to those observed in control culture and exert their immunomodulatory activity on effector T cells. GSD Treg cells preserved their inhibitory effects on the osteoclastogenesis. These results suggest that stimulation of Treg cells could open the way for the identification and testing of new therapeutic approaches for patients affected by GSD.

## Introduction

Gorham-Stout disease (GSD) or the so-called vanishing bone syndrome is a very rare disorder characterized by extensive bone resorption and vascular lesion. It was described for the first time in 1838 by Jackson who reported complete osteolysis of the humerus in a 12-year-old boy (Jackson, 1838). Fewer than 300 cases have been so far reported in the literature. GSD does not display a clear sex bias (1.6:1 male-to-female ratio) or inheritance pattern and can present at any age (age range is from 7 months to 80 years) (Patel, 2005; Dellinger et al., 2013, 2014; Hu et al., 2013; Nikolaou et al., 2014).

The disease may affect the appendicular or the axial skeleton (Patel, 2005; Dellinger et al., 2014). Nevertheless, Hu et al. (2013) recently reported a case series, in which the femur was the predominant affected bone. Radiologically, initial X-rays reveal changes resembling patchy osteoporosis (Nikolaou et al., 2014). At later stage bone deformity is observed with bone mass loss and concentric shrinkage in the long bones of upper and lower extremities. Eventually, a complete resorption of the bone occurs, resulting in the appearance of the so-called “vanishing bone” disease (Nikolaou et al., 2014). The most common symptoms of GSD are pain, swelling, weakness and functional impairment of affected regions. Moreover, approximately 25% of GSD patients develop chylothorax, which can result in respiratory distress and failure. Additionally, involvement of the vertebrae can cause severe neurological defects, deformity, paralysis and death (Dellinger et al., 2014).

The diagnosis of GSD is challenging and it is usually performed by exclusion criteria (Dellinger et al., 2013, 2014; Nikolaou et al., 2014). For the diagnosis, several investigations are used (radiographs, bone scan, and computed tomography), but the diagnosis must histologically confirmed revealing local bone progressive resorption, angiomatous tissue and absence of cellular atypia (Patel, 2005; Tolis et al., 2016).

Due to the rarity of the disease and to the limited studies, the etiopathogenesis is not entirely known. Indeed, preliminary studies by Prof. Lorenzo’s group suggested genetic imbalance in patients (Dellinger et al., 2013), but the pathways involved in the excessive bone erosion must be still identified. Nevertheless, the involvement of endothelial cells, osteoclasts, osteoblasts, and osteocytes has been suggested. Indeed, in GSD patients, increased serum levels of VEGF-A and -C have been found possibly stimulating lymphangiogenesis and osteoclastogenesis (Hagendoorn et al., 2014). Devlin et al. (1996) showed that patient serum can induce osteoclast formation in an IL-6-dependent manner. Recently, we demonstrated that Peripheral Blood Mononuclear Cells (PBMC) isolated from GSD patients showed increased ability to differentiate into osteoclasts with enhanced bone resorbing ability (Rossi et al., 2020). Moreover, we revealed defective osteoblast mineralization in patients (Rossi et al., 2020). Indeed, a remarkable aspect of GSD is the absence of bone formation activity by osteoblasts along surfaces of remaining bone fragments in sections of affected tissues (Dickson et al., 1990; Dellinger et al., 2014).

The involvement of immune system in the progressive osteolysis was recently suggested (Terashima and Takayanagi, 2018). It has been demonstrated that regulatory T cells (CD4^+^CD25^+^CD127^low^FoxP3^+^) are able to suppress the osteoclastogenesis while regulating osteoblast differentiation and activity (Zaiss et al., 2007; Del Fattore et al., 2010; Liu et al., 2011; Terashima and Takayanagi, 2018).

This study aimed to evaluate whether Treg could play an important role in the bone remodeling alterations leading to a complete bone loss in GSD patients.

## Materials and Methods

### Patients

Ten patients were recruited by the Rare Diseases and Medical Genetics Unit of Bambino Gesù Children’s Hospital (OPBG). The description of nine patients was previously reported (Rossi et al., 2021). A 12 years old female patient with swelling of the frontal bone, osteolysis of frontal and parietal bone, vertebrae and left scapula has been recently recruited for this study (Supplementary Table 1). Informed consent was obtained by parents/legal guardian. The study was approved by OPBG ethical committee (Protocol number: GR-2019-12370244, 01/02/2021). The diagnosis of GSD was based on radiological assessment and bone histology. Healthy Donor (HD) subjects were matched for age and gender and tested for C-Reactive Protein and Erythrocyte Sedimentation Rate to exclude inflammatory status.

### ELISA Assay

Peripheral blood samples from 7 patients and 21 HD were centrifuged 1,000 g × 10 min at room temperature and serum was collected and stored at –80°C. Levels of TGFβ1 (Transforming Growth Factor-β1; R&D Systems, Minneapolis, United States), IL-4 (Interleukin 4; R&D Systems, Minneapolis, United States), IL-6 (R&D Systems, Minneapolis, United States), and IL-10 (R&D Systems, Minneapolis, United States) were measured by ELISA kits, according to the manufacturers’ instructions.

### Peripheral Blood Mononuclear Cells Isolation

Peripheral Blood Mononuclear Cells from 6 patients and 14 HD were prepared from peripheral blood layered over Ficoll 1.077 g/ml (PANCOLL, PAN Biotec, Germany) and centrifuged at 400 g for 30 min. “Buffy coat” was collected and washed twice with Phosphate Buffered Saline (PBS, Euroclone, Milano, Italy). Cells were resuspended in Dulbecco’s Modified Essential Medium (DMEM, Euroclone, Milano, Italy) containing 50 U/ml penicillin, 50 mg/ml streptomycin, 2 mM L-glutamine, and 10% FBS (Fetal Bovine Serum).

### Flow Cytometry Analysis

PBMC isolated from five patients and eight HD were incubated in the dark for 20 min at 4°C with directly conjugated monoclonal antibodies, purchased from BD Biosciences, directed against the following human surface molecules: CD3 (1:40 Alexa Fluor 700-conjugated; clone UCHT1), CD4 (1:5 PerCP-Cy 5.5 conjugated; clone L200), CD8 (1:90 APC-Cy7 conjugated; clone SK1), CD25 (1:5 PE-conjugated; clone 2A3) and CD127 (1:5 Alexa Fluor 647 conjugated; clone 40131.111). After labeling, cells were washed in PBS and analyzed with BD LSRFortessa^TM^ FACS. CD4^+^/CD25^–^/CD127^high^ and CD4^+^/CD25^+^/CD127^low^ cells were considered effector T (Teff) cells and Treg, respectively (Del Fattore et al., 2015). Flow cytometry profiles were analyzed using FlowJo software (BD, San Jose, CA, United States).

### Histological/Histomorphometric Analysis of Bone Biopsies

Bone biopsies from lesions (L5 vertebral body, humerus, left ilium sacral region) of three pediatric patients and four HD were fixed in 10% formalin and processed for paraffin embedding with previous decalcification. For immunohistochemical analysis, sections were labeled with antibodies against human FoxP3 (Clone number 236A/E7) (Abcam, Cambridge, United Kingdom) at 4°C overnight, followed by secondary incubation for 1 h at room temperature with the corresponding secondary antibody (Agilent, Santa Clara, CA, United States). Histomorphometric measurements were carried out on 2–5 micron thick sections, with an interactive image analysis system (NIS-Elements BR 4.50.00). The Eroded Surface/Bone surface was evaluated according to the guidelines suggested by the Histomorphometry Nomenclature Committee of the American Society for Bone and Mineral Research (ASBMR) (Dempster et al., 2013). In ∼2.1 × 10^5^ μm^2^ of the ROI analyzed, FoxP3^+^ cells in the bone marrow were counted and data were reported as positive cells per bone marrow area (mm^2^).

### Stimulation of Treg Cells

Peripheral Blood Mononuclear Cells (PBMC) were labeled with 0.5 μM Carboxyfluorescein Succinimidyl Ester (CFSE, Thermo Fischer Scientific, Waltham, MA, United States) according to manufacturer’s instructions and cultured in RPMI-1640 medium (Euroclone, Milano, Italy) supplemented with 10% FBS with or without T-cell stimulation for 5 days. T stimulation was achieved adding Dynabeads Human T-activator CD3/CD28 beads (Thermo Fischer Scientific, Waltham, MA, United States) at a ratio of 1:50 (Del Fattore et al., 2015). Five-day non-adherent cells were resuspended, washed in PBS and analyzed by fluorescent-activated cell sorting (FACS) analysis.

### Osteoclast and Treg Co-culture

Peripheral Blood Mononuclear Cells (PBMC) from three healthy donors were isolated as previously described and 1 × 10^6^ cells/cm^2^ were plated on 96-well plates. After 3 h, cell cultures were rinsed to remove non-adherent cells. Cells were cultured in DMEM with 10% of FBS and in the presence of 20 ng/ml human Macrophage Colony-Stimulating Factor (hM-CSF, PeproTech, United Kingdom) and 30 ng/ml human Receptor Activator of NF-κB Ligand (hRANKL, PeproTech, United Kingdom) for 3 days. In the next medium changes, in addition to 50 μl of DMEM supplemented with FBS and hM-CSF/hRANKL, 2 × 10^4^ sorting-purified CD4^+^/CD25^+^/CD127^low^ cells (from six HD and three patients) in 50 μl RPMI-1640 were added per well. As controls, cells were treated with 50 μl of DMEM supplemented with FBS and hM-CSF/hRANKL, added with 50 μl RPMI-1640. After 10 days, cells were fixed in 4% paraformaldehyde and stained for Tartrate Resistant Acid Phosphatase (TRAcP, Sigma-Aldrich, United States). Osteoclasts were identified as TRAcP^+^ multinucleated (≥3 nuclei) cells. Leica DMi8 microscope and LasX software were used for cell analysis.

### Conditioned Medium Preparation

Peripheral Blood Mononuclear Cells (PBMC) (1 × 10^6^ cells/ml) from patients and HD were maintained in culture with complete medium (RPMI-1640, 10% FBS, 50 U/ml penicillin, 50 mg/ml streptomycin) with or without aCD3/CD28 beads. After 5 days, medium was collected and beads were removed using a magnet; supernatants were centrifuged 500 g for 5 min to remove cell contamination and stored at –80°C.

### Osteoclast Precursor Treatment With Conditioned Media

PBMC cultured for 3 days with 20 ng/ml hM-CSF and 30 ng/ml hRANKL were treated with (i) complete medium + 20 ng/ml hM-CSF + 30 ng/ml hRANKL, (ii) 100% Conditioned Medium (CM) from 5-day aCD3/CD28 stimulated PBMC of HD + 20 ng/ml hM-CSF + 30 ng/ml hRANKL, (iii) 100% CM from 5-day aCD3/CD28 stimulated PBMC of patients + 20 ng/ml hM-CSF + 30 ng/ml hRANKL. After 48 h total RNA was extracted from treated cells.

### Osteoclastogenic Assay With CM

One million/cm^2^ of HD PBMC were seeded in a 96-well culture plate. After 3 h non-adherent cells were removed and cultures were maintained under three different conditions: (i) complete medium + 20 ng/ml hM-CSF + 30 ng/ml hRANKL, (ii) 100% CM from 5-day aCD3/CD28 stimulated PBMC of healthy donors + 20 ng/ml hM-CSF + 30 ng/ml hRANKL, (iii) 100% CM from 5-day aCD3/CD28 stimulated PBMC of patients + 20 ng/ml hM-CSF + 30 ng/ml hRANKL. Medium was changed every 3 days. After 14 days, cells were stained for TRAcP and analyzed by microscopy.

### Bone Resorption Assay With CM

Three HD-PBMC were differentiated on bovine bone slices (IDS, PANTEC) for 2 weeks and then treated for 4 days with (i) complete medium + 20 ng/ml M-CSF + 30 ng/ml RANKL, (ii) 100% CM from 5-day aCD3/CD28 stimulated PBMC of 3 HD + 20 ng/ml M-CSF + 30 ng/ml RANKL, (iii) 100% CM from 5-day aCD3/CD28 stimulated PBMC of 3 patients + 20 ng/ml M-CSF + 30 ng/ml RANKL. Then, cells were removed by prolonged sonication and section were stained with 1% toluidine blue. Resorption pits were observed by conventional light microscopy and area was measured by image analysis system (NIS Elements BR 4.50.00).

### Real-Time RT-PCR

Total RNA was extracted using TRI Reagent (Sigma-Aldrich, United States); one microgram of RNA was reverse transcribed (SensiFAST cDNA synthesis kit, Bioline, United Kingdom) in a volume of 20 μl to produce complementary DNA (cDNA) and 25 ng of cDNA was used for Real-Time PCR reactions using SensiFAST SYBR Low-ROX kit (Bioline, United Kingdom). Primer sequences are listed in Table 1.

**TABLE 1:** List of primer pairs and sequence.

Gene	Forward primer	Reverse primer
*DC-STAMP*	5′-GGACATGGCTGGGACTGAAA-3′	5′-TGTTCTGCTGTGTTGCTCCA-3′
*ATP6V0D2*	5′- GGTCTCTCGGTCTTCTTTGC-3′	5′-CCTTGGGCCGTTTCACAGAA-3′
*CLC7*	5′-TGATCTCCACGTTCACCCTGA-3′	5′-TCTCCGAGTCAAACCTTCC-3′
*TCRG1*	5′-GGGATCCAGGGTAAGCATCG-3′	5′-CCGCTCCCTACACCATCATC-3′
*MMP9*	5′-TTGACAGCGACAAGAAGTGG-3′	5′-GCCATTCACGTCGTCCTTAT-3′
*CATK*	5′-TCGGGGATCTCTCTGTACCC-3′	5′-CCCGCAGTAATGACACCCTT-3′
*GAPDH*	5′-GACAAGCTTCCCGTTCTCAG-3′	5′-ACAGTCAGCCGCATCTTCTT-3′

### Statistics

Data were expressed as the mean ± SD of at least three independent experiments. Statistical analysis was performed by one-way analysis of variance, followed by the unpaired Student’s *t*-test or the Mann-Whitney *U* test using GraphPad Prism 5. A *p*-value ≤ 0.05 was considered statistically significant.

## Results

### Serum Analysis

Serum analysis of IL-4, IL-6, IL-10, and TGFβ1 was performed to evaluate alteration of cytokines involved in Treg regulation and activity. As shown in Table 2, high levels of IL-6 and reduction of TGFβ1 (Transforming Growth Factor-β) were revealed in GSD patients (Table 2). No detectable levels of IL-4 were measured (data not shown) and no differences of IL-10 values were observed between GSD patients and age- and sex-matched HD (Table 2).

**TABLE 2:** Serum cytokines involved in Treg regulation and activity.

	HD	GSD	p
IL-6 (pg/mL)	0.10 ± 0.05	1.89 ± 1.00*	0.001
IL-10 (pg/mL)	12.11 ± 4.77	4.51 ± 1.89	0.317
TGFβ1 (pg/mL)	27,680 ± 1,131	22,830 ± 2,509*	0.050

Results are expressed as mean ± SD. *p ≤ 0.05.

### Immunomodulatory Activity of GSD Treg

**FIGURE 1:**
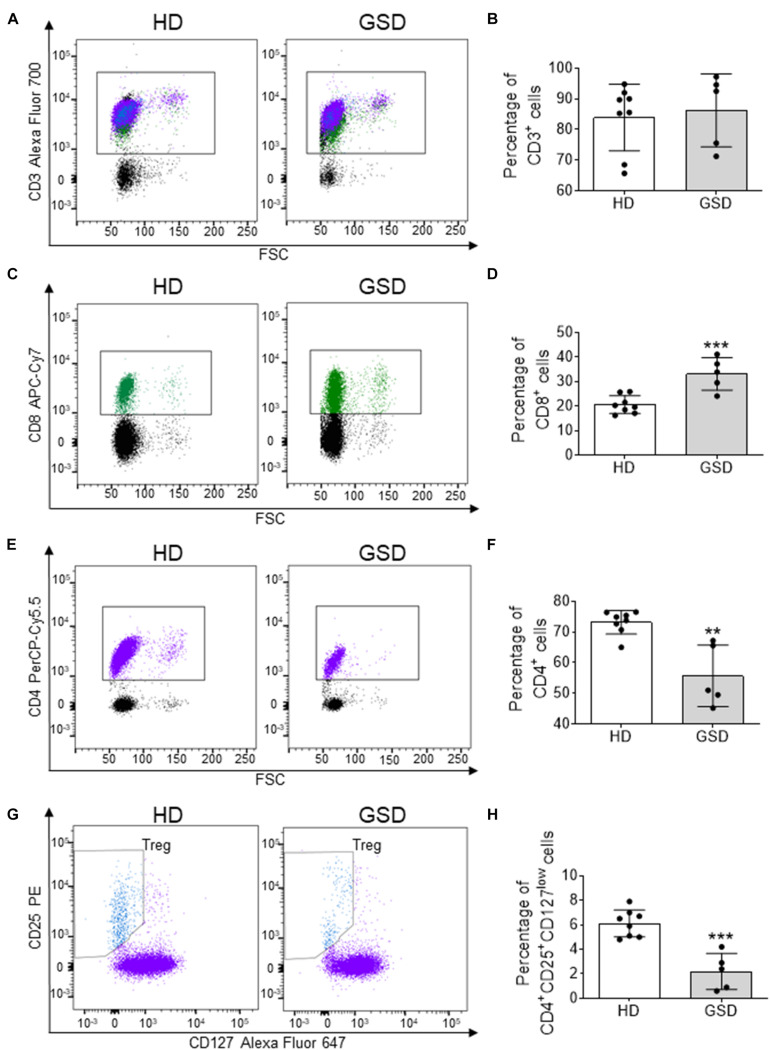
Cytometric analysis performed on PBMC isolated from five patients and eight HD. **(A)** Representative FACS plot for CD3^+^ cells; CD8^+^ cells are in green, while CD4^+^ and T reg are marked in purple and blue, respectively. **(B)** Percentage of CD3^+^ cells. **(C)** Representative FACS analysis for CD8^+^ cells in CD3^+^ gate. **(D)** Percentage of CD8^+^ cells in CD3^+^ gate. **(E)** Representative FACS plot for CD4^+^ cells. **(F)** Percentage of CD4^+^ cells in CD3^+^ gate. **(G)** Representative FACS analysis for CD25^+^CD127^low^ cells in CD4^+^ gate. **(H)** Percentage of CD25^+^CD127^low^ Treg in CD4^+^ gate. Results are expressed as mean ± SD. **p < 0.01; ***p < 0.001.

To evaluate alterations of T cells in GSD, PBMC were isolated from patients and HD and they were analyzed using flow cytometry. No alteration in CD3 population (Figures 1A,B) was identified in GSD patients, while an increase in CD8^+^ subpopulation (Figures 1C,D) and a reduction of CD4^+^ cells (Figures 1E,F) were disclosed. In CD4^+^ cells gate, Treg were identified by expression of CD25 and low level of CD127. As shown in Figures 1G,H Treg were 2.2% of total CD4^+^ T cells in peripheral blood of patients (range 0.6–4.2%) and 6.1% in HD (range 4.8–7.9%). Therefore, the percentage of Treg was significantly decreased compared to HD (*p* = 0.0002), without difference of Teff [% of CD4^+^CD25^–^CD127^high^ in CD4^+^ gate (mean ± SD): HD: 52.31 ± 11.17; GSD: 53.36 ± 19.67; *p* = 0.90].

**FIGURE 2:**
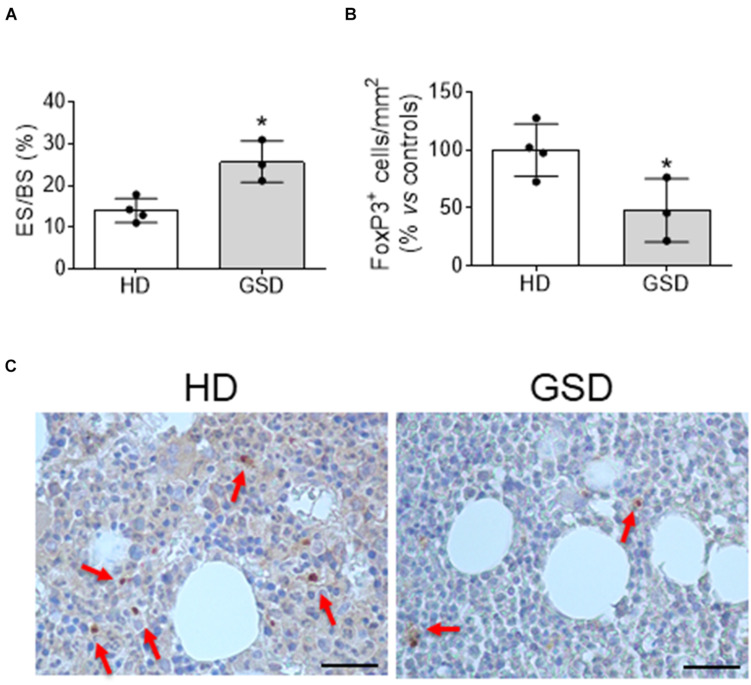
Bone biopsy analysis of four HD and three patients. **(A)** Histomorphometric analysis of Eroded surface/Bone surface (%). **(B)** Histomorphometric analysis of FoxP3^+^ cells/Total Bone marrow Area. **(C)** Representative pictures of immunohistochemical analysis of FoxP3. The arrows indicate FoxP3^+^ cells. Scale bar: 25 μm. Results presented as percentage vs. aged-matched HD. Results are expressed as mean ± SD. *p < 0.05.

The reduction of Treg was also confirmed in bone microenvironment by immunohistochemical analysis of bone biopsy. Indeed, besides the increased bone erosion (Figure 2A) histomorphometric measurement revealed about 50% reduction of FoxP3^+^ cells in bone marrow of three patients compared to HD (Figures 2B,C).

**FIGURE 3:**
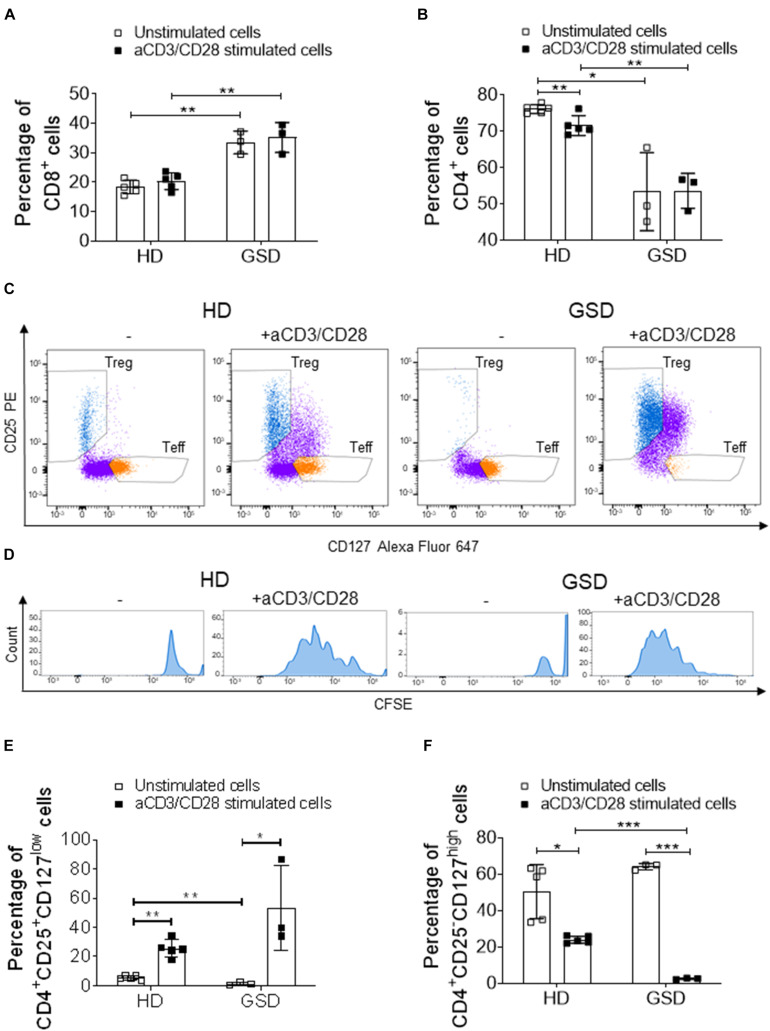
Cytometric analysis performed on PBMC, isolated from three patients and five HD, stimulated or not with aCD3/CD28 beads for 5 days. **(A)** Percentage of CD8^+^ cells and **(B)** CD4^+^ cells in CD3^+^ gate. **(C)** Representative FACS plots of HD and GSD CD4^+^ cells with or without anti-CD3/CD28 (CD4^+^ cells are marked in purple, while T reg and Teff are reported in blue and orange, respectively). **(D)** Representative plots of CFSE^+^-labeled proliferative Treg (marked in blue) of HD and GSD patient stimulated or not with aCD3/CD28 beads. **(E)** Percentage of CD25^+^CD127^low^ Treg in CD4^+^ gate. **(F)** Percentage of CD25^–^CD127^high^ Teff in CD4^+^ gate. Results are expressed as mean ± SD. *p < 0.05; **p < 0.01; ***p < 0.001.

Next, we evaluated the proliferation ability of CD4^+^CD25^+^CD127^low^ Treg incubating PBMC with anti-CD3/anti-CD28-coated microbeads. This polyclonal expansion protocol greatly specifically increases Treg number while preserving their suppressive capacity (Del Fattore et al., 2015). Indeed, HD and patient CD8^+^ cells and GSD CD4^+^ were not affected by treatment (Figures 3A,B); the stimulation induced a slight decrease of HD CD4^+^ subpopulation (Figure 3B). Anti-CD3/anti-CD28 (aCD3/CD28) beads induced the proliferation of Treg (Figures 3C–E); surprisingly, GSD cultures had a higher proliferation rate compared to HD as shown by FACS analysis revealing increased CFSE dilution for the multiple generations (Figure 3D). Moreover, a significant reduction of CD4^+^CD25^–^CD127^high^ (Teff) number was revealed in stimulated cultures (Figures 3C,F), suggesting that GSD Treg maintain their immunomodulatory activity.

### Inhibition of Osteoclasts by Interaction With GSD Treg

**FIGURE 4:**
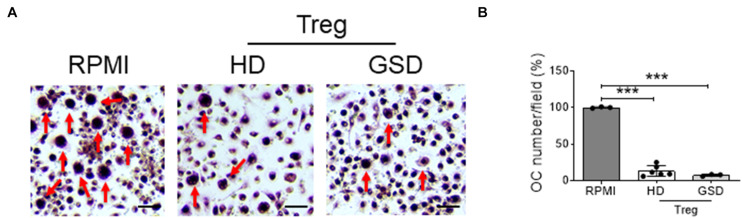
Peripheral Blood Mononuclear Cells (PBMC) were isolated from three HD, treated with hRANKL/hM-CSF and co-cultured with six HD and three GSD aCD3/CD28-stimulated and sorted Treg. As control, cells were treated with hM-CSF/hRANKL and RPMI-1640. **(A)** Representative picture of TRAcP staining. Scale bar: 75 μm. **(B)** Percentage of TRAcP positive multinucleated (>3 nuclei) cells compared to RPMI treated culture. Results are expressed as mean ± SD. ***p < 0.001.

**FIGURE 5:**
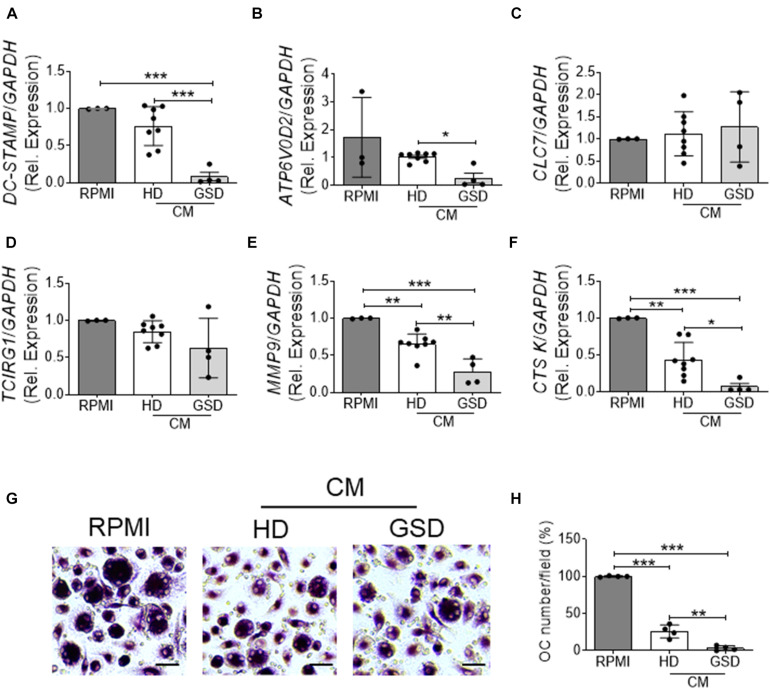
**(A–F)** RNA was extracted from three HD-osteoclast precursors treated for 48 h with RPMI-1640 + M-CSF/RANKL as control (RPMI group) or 100% conditioned medium (CM) of aCD3/CD28 stimulated PBMC isolated from four patients and eight HD + hM-CSF/hRANKL and reverse transcribed; then cDNA was subjected to comparative Real-Time PCR using primer pairs and specific conditions for **(A)** *DC-STAMP*, **(B)** *ATP6V0D2*, **(C)** *CLC7*, **(D)** *TCIRG1*, **(E)** *MMP9*, and **(F)** *CTSK* genes. qRT-PCR Cycle threshold ranges are *DC-STAMP*: 19.5–28.2; *ATP6V0D2*: 22.6–29.9; *CLC7*: 23.0–27.0; *TCIRG1*: 21.8–26.6; *MMP9*: 18.0–22.6; *CTSK*: 20.9–28.6; *GAPDH*: 18.0–19.6. **(G,H)** PBMC were isolated from four HD and treated with RPMI-1640 + hM-CSF/hRANKL as control (RPMI group) or 100% conditioned medium (CM) of aCD3/CD28 stimulated PBMC isolated from four patients and four HD + hM-CSF/hRANKL. **(G)** Representative pictures of TRAcP staining. Scale bar: 30 μm. **(H)** Percentage of TRAcP positive multinucleated (>3 nuclei) cells. Results are expressed as mean ± SD. *p < 0.05; **p < 0.01; ***p < 0.001.

To evaluate the effects of Treg on osteoclast differentiation, we co-cultured hRANKL/hM-CSF-treated PBMC isolated from healthy donors with Treg cells of GSD patients and HD. A reduction of about 70–80% of TRAcP-positive multinucleated cells was observed in cultures of PBMC with Treg (Figures 4A,B); GSD cells retained their inhibitory activity on osteoclastogenesis (Figures 4A,B). No differences were revealed in the number of nuclei/osteoclast [N° nuclei/osteoclast (mean ± SD): PBS: 3.48 ± 0.46; HD Treg: 3.06 ± 0.11; GSD Treg: 3.00 ± 0.01]. To evaluate whether these effects were dependent on the direct cell-cell contact, we treated HD PBMC with conditioned medium isolated from aCD3/CD28-stimulated PBMC of healthy donors and patients. Real Time RT-PCR expression analysis revealed that the treatment for 48 h with GSD cell conditioned medium induced a downregulation of the osteoclast fusion gene *DC-STAMP* (Dendritic Cell-Specific TrAnsMembrane Protein) (Figure 5A) in osteoclast precursors, compared to cells treated with osteoclastogenic medium and with HD cells-derived conditioned medium, respectively. Conditioned medium from GSD cells induced a downregulation of *ATP6V0D2* (ATPase H + Transporting V0 Subunit A2) (Figure 5B) expression compared to cells treated with HD cells medium while no significant differences were observed between PBMC treated with osteoclastogenic medium and CM (*p* = 0.08).

No alterations were observed in the expression of genes involved in the acidification of resorption lacunae including *CLC7* (Chloride Channel Type 7) (Figure 5C) and *TCIRG1* (T Cell Immune Regulator 1, ATPase H+ Transporting V0 Subunit A3) (Figure 5D). The treatment with conditioned medium downregulated the expression of *MMP*9 (Metalloprotease Type IX) and *CTSK* (Cathepsin K) important for the resorption of bone matrix; CM of cells isolated from patients induced a stronger ability to reduce the expression of these genes compared to that derived from HD cells (Figures 5E,F).

To investigate the mechanisms of inhibition, we evaluated the effects of conditioned medium on osteoclastogenesis and on bone resorption activity. As shown in Figures 5G,H, ∼75% reduction of osteoclast formation was observed in cultures treated with CM from HD’ cells as revealed by TRAcP staining (Figure 5G) and the quantification of multinucleated (>3 nuclei) TRAcP positive cells (Figure 5H). The conditioned medium from GSD cultures was about 7.5-fold more effective than that from HD cells in inhibiting osteoclast differentiation (Figures 5G,H). No alteration was observed in the number of nuclei per cell (Number of nuclei/osteoclast; RPMI: 3.53 ± 0.39; HD CM: 3.07 ± 0.07; GSD CM: 3.00 ± 0.06) and in the resorption ability (Resorbed Area/Total Area, %; RPMI: 4.03 ± 1.02; HD CM: 4.19 ± 1.09; GSD CM: 3.70 ± 1.58).

## Discussion

**FIGURE 6:**
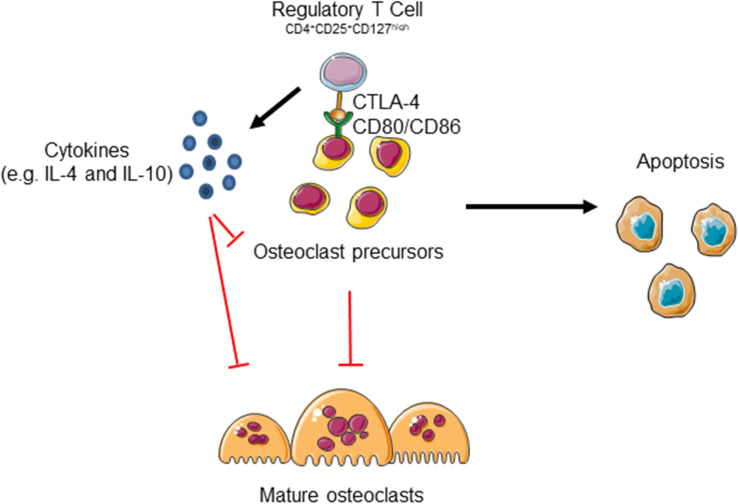
Regulatory T cells could inhibit osteoclast formation by two mechanisms. Treg cells release several cytokines including IL-4 and IL-10 that inhibit osteoclast formation. Moreover, the binding of Cytotoxic T Lymphocyte-associated antigen 4 (CTLA-4) to CD80/CD86 receptor expressed on osteoclast precursors (pOC) induces pOC’ apoptosis. The figure was produced using Servier Medical Art.

Gorham Stout disease is a very rare disorder characterized by progressive bone erosion and vascular lesion. In this study we evaluated for the first time the role of immune cells in the progressive osteolysis. Indeed, the immune system and bone are tightly regulated and the term osteoimmunology was coined by Arron and Choi to describe the association between osteoclasts and T cells (Arron and Choi, 2000). These cells share the same hemopoietic origin and, as other hematopoietic cells, osteoclast precursors can be detected in the peripheral blood. Moreover, many inflammatory diseases are characterized by increased bone erosion including Rheumatoid Arthritis and Periodontal Disease (Sato et al., 2013). In GSD patients we observed an increase of bone resorbing activity as shown in Figure 2A and high levels of CD8^+^ cells that could be related to the increase of osteoclastogenesis that we reported in our previous study (Rossi et al., 2020). Indeed, Kiesel et al. (2009) showed that murine osteoclasts are able to induce CD8^+^ T cell proliferation and activation by antigen cross-presentation. Interestingly, a reduction of CD4^+^ cells was observed in our patients. It was proved that osteoclasts are able to interact with naïve CD4^+^ cells and to induce FoxP3^+^ CD4^+^ T cells in an antigen-specific manner (Ibanez et al., 2016). Interestingly, in the bone marrow Tregs localize within 15 μm of the endosteal surface (Fujisaki et al., 2011). Regulatory T cells can inhibit bone erosion by the release of inhibitor cytokines or by direct cell-cell contact (Figure 6). Zaiss et al. (2007) demonstrated that regulatory CD4^+^CD25^+^Foxp3^+^ T cells suppress osteoclast formation in a cell contact-dependent manner, and that bone resorption serum markers inversely correlate with the amount of circulating Treg. The cell contact inhibition is mediated by T lymphocyte-associated antigen 4 (CTLA4) that directly binds to CD80/CD86 on osteoclast precursors’ surface inducing cell apoptosis and inhibiting mature osteoclast formation (Figure 6; Bozec et al., 2014). Further studies showed that Treg cells protect from local and systemic bone destruction (Yuan et al., 2010; Zaiss et al., 2010). In our GSD patients we observed a reduction of CD4^+^CD25^+^CD127^low^ cells in peripheral blood and of FoxP3^+^ cells in the bone marrow. Furthermore, we demonstrated that GSD CD4^+^CD25^+^CD127^low^ Treg maintain their immunomodulatory activity, as clarified by their ability to proliferate with aCD3/CD28 treatment and their immunosuppressive ability against Teff cells. For the isolation of Treg cells, we used surface markers CD4/CD25/CD127 since FoxP3 is an intracellular factor and its analysis requires permeabilization of the cells (Liu et al., 2006; Del Fattore et al., 2015). We evaluated their interplay with osteoclast precursors and osteoclasts and we established that patient stimulated Treg are able to inhibit osteoclastogenesis by co-culture experiments and treatment with conditioned medium.

In the interplay between bone and immune system cytokines and secreted factors play a crucial role. Factors with regulatory properties in inflammatory responses, such as TGFβ1, IL-4 and IL-10, also negatively regulate osteoclast formation and bone resorption. Since it was demonstrated that interleukin-6 inhibits regulatory T cells (Lin et al., 2012) and TGFβ is essential for signals in safe-guarding specific Treg cell functions (Konkel et al., 2017), we performed serum analysis that revealed increase of IL-6, reduction of TGFβ1, and no significant alteration for IL-10. It was also evident that sclerostin promotes the production of IL-6 and transforming growth factor β in mesenchymal stem cells, reducing the differentiation of Treg (You et al., 2018). In our previous paper, we detected high levels of sclerostin in serum samples from GSD patients that could be correlated to defective bone formation and Treg alterations (You et al., 2018). Indeed, You et al. (2018) demonstrated that sclerostin promotes the differentiation of Th17 cells and reduces the differentiation of Treg. Concerning the interplay between Treg and osteoblast lineage, Liu et al. (2011) showed a positive effect on healing upon administration of combined Treg and bone marrow mesenchymal stem cells in a calvarial defect model in mice.

Due to rarity of the disease, limited published studies and incomplete knowledge of the etiology, the GSD lacks effective therapeutic approaches. Available therapeutic options (anti-VEGF-A antibody, bisphosphonates, radiation) are not curative and can be associated with severe side effects (Hagendoorn et al., 2014). The understanding of the impact of immune system on bone remodeling alterations observed in GSD patients could contribute to the identification of new therapeutic options for this rare disease. Recently, there has been an explosion in research investigating the potential to manipulate Treg for clinical purposes (Trzonkowski et al., 2009; Marek-Trzonkowska et al., 2012; von Boehmer and Daniel, 2013). There are several phase I clinical trials to test whether boosting Treg numbers and/or function is a feasible, safe, and potentially effective way to treat diseases such as graft vs. host disease, type 1 diabetes and to prevent the rejection of transplanted organs (Trzonkowski et al., 2009; Marek-Trzonkowska et al., 2012; von Boehmer and Daniel, 2013). An increase of the number of Treg is achievable by Treg transfer or treatment with mTOR (mammalian target of rapamycin) inhibitors (von Boehmer and Daniel, 2013). Recent studies (Ozeki and Fukao, 2019; Ricci et al., 2019; Liang et al., 2020) reported that effectiveness of mTOR inhibitor Sirolimus in the treatment of GSD patients; indeed Sirolimus is known to inhibit lymphangiogenesis and is thought to act on lymphatic tissue within lesions regulating production and leakage of lymph (Ozeki and Fukao, 2019). Sirolimus also stimulates Treg (Battaglia et al., 2006; Strauss et al., 2009), that according with our results, could inhibit bone erosion. Further studies are required to elucidate the effects of Sirolimus treatment on GSD immune and bone cells. However, these observations strengthen the concept that targeting the mechanisms involved in the immune system-bone crosstalk could represent a promising therapeutic approach for GSD patients.

## Data Availability Statement

The original contributions presented in the study are included in the article/Supplementary Material, further inquiries can be directed to the corresponding author/s.

## Ethics Statement

The studies involving human participants were reviewed and approved by the OPBG Ethical Committee (Protocol Number: GR-2019-12370244, 01/02/2021). Written informed consent to participate in this study was provided by the participants’ legal guardian/next of kin.

## Author Contributions

MR performed the osteoclast culture. GB and VD contributed to the gene expression analysis. IR, PSB, DV, MVG, and AJ recruited the patients and performed the ELISA assays. OP, MD’A, CC, and SM contributed to the hematologic tests and discussion of the results. RD performed the histological analysis. ADF and AB designed and supervised the work and wrote the manuscript. ADF was the guarantor of this work and, as such, had full access to all of the data in the study and takes responsibility for the integrity of the data and the accuracy of the data analysis. All authors reviewed the manuscript and approved the final version.

## Conflict of Interest

The authors declare that the research was conducted in the absence of any commercial or financial relationships that could be construed as a potential conflict of interest.

## Publisher’s Note

All claims expressed in this article are solely those of the authors and do not necessarily represent those of their affiliated organizations, or those of the publisher, the editors and the reviewers. Any product that may be evaluated in this article, or claim that may be made by its manufacturer, is not guaranteed or endorsed by the publisher.
