# Supplementary material for: Stimulation of Treg Cells to Inhibit Osteoclastogenesis in Gorham-Stout Disease
Source: Front Cell Dev Biol. 2021 Aug 27;9:706596. doi: 10.3389/fcell.2021.706596 (PMC8430039; doi:10.3389/fcell.2021.706596)
Supplement: Supplementary file 1 [file Table_1.DOCX]

**Supplementary Table 1.** Clinical findings of patients affected by Gorham-Stout disease.

| **Patient** | **Age** | **Sex** | **Clinical manifestations** |
| --- | --- | --- | --- |
| #1 | 17 | M | Osteolytic lesions of C5-T5-T6-T8-T9-L3-L4 vertebral bodies, sacrum, ribs, ilium and humerus  Angiomatous lesion of humerus |
| #2 | 7 | M | Severe osteolytic lesion of craniofacial and skull base  Large lymphatic cyst behind the ear that was removed at the age of 2  Relevant rotation of the craniocervical hinge |
| #3 | 10 | M | Disappearance of femoral diaphysis; Femur fracture  Formation of pseudo-cysts |
| #4 | 31 | F | Lesions of sternum, vertebral bodies, ilium and femurs  Inguinal swelling |
| #5 | 10 | M | Lesions of vertebral bodies, ribs, humerus and scapula  Chylothorax |
| #6 | 3 | M | Lesions of vertebral bodies, ribs, humerus and scapula  Chylothorax |
| #7 | 8 | M | Osteolysis of jaw  Pterygoid and parathyroid lymphangioma |
| #8 | 18 | M | Sphenoid bone and left mandible  Lymphangiomas, meningocele |
| #9 | 2 | F | Left mandible  Swelling of the left mandible |
| #10 | 12 | F | Osteolysis of frontal and parietal bone, vertebrae and left scapula  Swelling of the frontal bone |
